# Degree of Food Processing and the Risk of Immune-Mediated Inflammatory Diseases: A Prospective Analysis of the SUN Cohort

**DOI:** 10.3390/nu18121969

**Published:** 2026-06-18

**Authors:** Luiz Menezes-Júnior, Miguel A. Martinez-Gonzalez, Ainara Martínez-Tabar, Álvaro González-Cantero, Alejandro Martín-Gorgojo, Maira Bes-Rastrollo

**Affiliations:** 1 Post-Graduate Program in Health and Nutrition, Federal University of Ouro Preto, Ouro Preto 35400-000, Brazil; luiz.menezes@ufop.edu.br; 2 Department of Epidemiological Analysis and Surveillance of Non-Communicable Diseases, Ministry of Health, Brasilia 70058-900, Brazil; 3 Department of Preventive Medicine and Public Health, Faculty of Medicine, University of Navarra, 31008 Pamplona, Spain; amartinez.80@alumni.unav.es (A.M.-T.); mbes@unav.es (M.B.-R.); 4 IdiSNA, Navarra Institute for Health Research, 31008 Pamplona, Spain; 5 CIBER Fisiopatología de la Obesidad y Nutrición (CIBERobn), Instituto de Salud Carlos III, 28029 Madrid, Spain; 6 Department of Nutrition, Harvard T.H. Chan School of Public Health, Boston, MA 02115, USA; 7 Department of Dermatology, Hospital Universitario Ramon y Cajal, Instituto Ramón y Cajal de Investigación Sanitarian (IRYCIS), 28034 Madrid, Spain; alvaro.gonzalez@icmrresearch.com; 8 Faculty of Medicine, Universidad Francisco de Vitoria, 28223 Madrid, Spain; 9 Madrid City Council, STI/Dermatology Department, 28006 Madrid, Spain; alejandromartingorgojo@aedv.es

**Keywords:** ultra-processed foods, minimally processed foods, autoimmune diseases, cohort study, NOVA classification

## Abstract

Background and Objectives: The role of diet in the risk and clinical course of immune-mediated inflammatory diseases (IMIDs) is an area of ongoing research, in which prospective evidence regarding the degree of food processing is limited. We prospectively assess the association between ultra-processed food (UPF) and minimally or unprocessed food (MUPF) consumption and incident psoriasis, rheumatoid arthritis and vitiligo, as well as a composite exploratory IMID outcome, in a Spanish cohort. Methods: We followed 15,874 IMID-free participants from the SUN Project (median follow-up: 15.1 years). Baseline diet was assessed via a validated FFQ and categorized by NOVA classification. Multivariable Cox models estimated hazard ratios (HRs) and 95% confidence intervals (CIs) across consumption tertiles (% g/day). Results: During follow-up, 298 self-reported diagnosed incident IMID cases occurred (1.31/1000 person-years, mostly psoriasis and rheumatoid arthritis). After adjusting for sociodemographic, lifestyle, and clinical factors, the highest UPF consumption was associated with an increased risk of self-reported diagnosed psoriasis (T3 vs. T1: HR = 1.63, 95% CI: 1.08–2.45) and rheumatoid arthritis (T3 vs. T1: HR = 1.97, 95% CI: 1.19–3.26; *p*-trend = 0.006), whereas no significant association was observed for vitiligo. Similar trends were observed for the exploratory composite IMID endpoint (T3 vs. T1: HR = 1.80, 95% CI: 1.31–2.45). Conversely, higher MUPF intake was associated with a lower risk of self-reported diagnosed psoriasis (T3 vs. T1: HR = 0.60, 95% CI: 0.40–0.92; *p*-trend = 0.014) and exploratory composite IMID endpoints (T3 vs. T1: HR = 0.64, 95% CI: 0.47–0.87; *p*-trend = 0.003). Conclusions: Higher UPF consumption is associated with an increased risk of self-reported diagnosed psoriasis and rheumatoid arthritis, whereas MUPF intake appears to be inversely associated with self-reported diagnosed psoriasis risk. These findings contribute to the ongoing evidence regarding the degree of food processing as a potential factor in the epidemiological profile of specific autoimmune conditions. However, given the observational design of the study, these findings reflect longitudinal associations and do not imply causation.

## 1. Introduction

Immune-mediated inflammatory diseases (IMIDs) represent a significant and growing global health challenge, characterized by a breakdown of immunological tolerance resulting in a disruption of immune regulation that leads to damage of autologous organs and tissues. This process results in chronic inflammation and often irreversible functional impairment [1,2]. While genetic predisposition provides the framework for IMIDs, the rapid increase in the incidence of these diseases in industrialized nations over recent decades strongly implicates environmental and lifestyle factors as key drivers [2,3]. While the role of diet and specific dietary patterns in the risk and clinical course of IMIDs is currently an area of ongoing investigation, nutrition has emerged as a critical area of scientific interest, recognized not merely for its role in sustenance but as a dynamic and complex modulator of immune homeostasis capable of influencing physical barriers, the gut microbiome, and both innate and adaptive immune responses [4].

The clinical relevance of dietary patterns in IMIDs is supported by a growing body of evidence across different conditions. For instance, high adherence to a Mediterranean diet has been associated with a lower risk of developing rheumatoid arthritis [5] and Crohn’s disease [6], while specific interventions have shown significant reductions in disease severity for patients with psoriasis [7] and ulcerative colitis [8,9]. The concept that diet plays a central role in immune regulation is further supported by the fact that a significant portion of the immune system is localized within the gastrointestinal tract, where it must constantly interact with the vast antigenic load introduced through food.

Concurrently, the rise of the Western diet, characterized by high intake of saturated fats, cholesterol, refined sugars, and excess salt intake, has been epidemiologically correlated with the increasing prevalence of various IMIDs. However, much of the existing nutritional research on these conditions has focused on isolated nutrients or broad dietary patterns [10,11]. The degree of industrial food processing itself is now recognized as a critical dimension of diet quality, independent of nutrient composition. The NOVA classification system, which categorizes foods by the nature, extent, and purpose of processing (e.g., ultra-processed foods vs. minimally processed/unprocessed foods), provides a novel framework for investigation. Emerging research is applying this framework to IMIDs. A recent large prospective cohort study found that higher consumption of ultra-processed foods (UPFs) was associated with a greater than 50% increased risk of developing systemic lupus erythematosus, with the risk doubling for a specific, more severe subtype [12]. Similarly, a meta-analysis has indicated that a pro-inflammatory diet, often high in processed foods, is associated with an increased likelihood of developing multiple sclerosis and other demyelinating diseases [13]. These findings underscore the potential importance of food processing as a key environmental exposure. However, evidence remains scarce, particularly for other common IMIDs like rheumatoid arthritis, psoriasis, and vitiligo and for the potential protective role of diets high in minimally processed and unprocessed foods (MUPFs).

Therefore, the primary objective of the present study was to prospectively investigate the associations between the consumption of UPFs and MUPFs, classified according to the NOVA system, and the risk of specific incident IMIDs (psoriasis, rheumatoid arthritis, and vitiligo) in a large cohort of Spanish university graduates. As a secondary and exploratory objective, we examined these associations for a composite IMID endpoint. By examining these associations for the exploratory IMID composite outcome and for specific diseases, this research aims to fill a significant gap in the literature and provide new insights into how modifiable dietary factors, defined by their level of processing, may influence the development of autoimmune conditions.

## 2. Materials and Methods

### 2.1. Study Population and Design

The SUN (Seguimiento Universidad de Navarra) is a multipurpose, prospective, and dynamic cohort study designed to assess the relationship between dietary patterns, lifestyle factors, and chronic disease incidence among Spanish university graduates. Initiated in December 1999, the cohort employs an open, rolling recruitment strategy [14,15,16]. Participants are Spanish graduates who are invited to participate via mail or email. Eligibility requires having completed a university degree and being aged 20 or older at enrollment. Participation is voluntary and not incentivized.

The study protocol was approved by the Human Research Ethical Committee at the University of Navarra (approval code: 2001/30, 30 August 2001), and all participants provided informed consent by completing the baseline questionnaire. Although the study is observational in nature, it was registered in ClinicalTrials.gov to enhance transparency and methodological rigor (identifier: NCT02669602; 27 January 2016). The study adheres to the principles of the Declaration of Helsinki.

**Figure 1:**
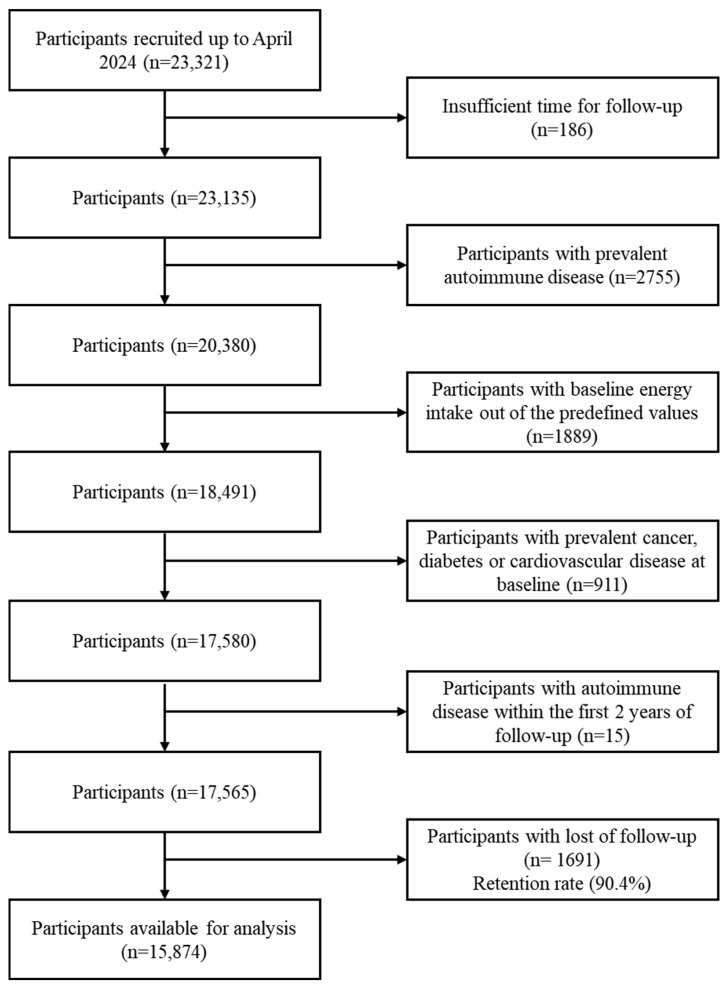
Flowchart of participants in the study. The SUN Project.

Upon enrollment, participants completed a comprehensive baseline questionnaire (Q0) assessing sociodemographic characteristics, medical history, lifestyle habits, and dietary intake [14]. Follow-up questionnaires are distributed biannually to update information on lifestyle, dietary changes, and the occurrence of new medical diagnoses. Figure 1 illustrates the participant selection process. From the 23,321 participants recruited in the SUN Project up to April 2024, we initially excluded 186 individuals who did not have enough time to be followed up, leaving 23,135 participants. We then excluded 2755 participants with prevalent IMIDs at baseline. After further excluding 1889 participants with total energy intake values outside the predefined ranges proposed by Willett (<500 or >3500 kcal/day for women and <800 or >4000 kcal/day for men), 18,491 participants remained. Subsequently, 911 participants with prevalent cancer, diabetes, or cardiovascular disease at baseline were excluded, and 15 participants who developed IMIDs within the first two years of follow-up were also excluded to minimize reverse causality. Finally, after excluding 1691 participants lost to follow-up (retention rate: 90.4%), the analytical sample comprised 15,874 participants free of IMIDs, cancer, diabetes, and major cardiovascular disease at baseline.

### 2.2. Outcome: Immune-Mediated Inflammatory Disease Incidence

The primary focus of this study was the self-reported diagnosed incidence of three specific reported conditions, rheumatoid arthritis, psoriasis, and vitiligo, as these represent the most prevalent IMIDs systematically assessed within the SUN Project follow-up protocols. Additionally, we evaluated a secondary, exploratory composite endpoint comprising these three IMIDs. Reframing the specific diseases as the primary focus accounted for their distinct pathophysiologies, while the exploratory composite endpoint allowed for an assessment of shared inflammatory pathways with greater statistical power.

Incident cases were identified through self-reports in follow-up questionnaires, where participants were asked if a doctor had ever diagnosed them with specific conditions, including “rheumatoid arthritis,” “psoriasis,” and “vitiligo.” For each reported case, participants were asked to provide the date (month and year) of diagnosis. This self-reported diagnosis method has been validated and widely used in the SUN cohort and other large epidemiological studies for various chronic conditions [17,18]. For participants who reported more than one IMID, the date of the first diagnosed event was used in the analysis of the composite event.

### 2.3. Assessment of Dietary Exposure

Dietary intake was assessed at baseline and repeated during follow-up at 10 and 20 years using a validated, self-administered 136-item semi-quantitative food frequency questionnaire (FFQ). This FFQ has been validated in the Spanish population and shows adequate reproducibility [19]. For each food item, participants reported their average frequency of consumption over the previous year using nine categories ranging from “never or rarely” to “≥6 times per day.” Standard portion sizes were specified, and nutrient intake was calculated using an updated Spanish food composition database [20,21].

Food items from the FFQ were classified according to the NOVA food processing classification system, which categorizes foods based on the extent and purpose of industrial processing in group 1, MUPF (e.g., fresh fruits, vegetables, legumes, meat, fish, eggs, and milk); group 2, processed culinary ingredients (e.g., oils, butter, sugar, and salt); group 3, processed foods (e.g., canned vegetables, cheeses, and fresh bread); and group 4, UPF (e.g., sugar-sweetened beverages, processed meats, industrial baked goods, and ready-to-eat meals) [22]. The primary exposures of interest in this study were the consumption of group 1 (MUPF) and group 4 (UPF), as presented in the Supplementary Materials (Supplementary Table S1). For each participant, we calculated the total daily grams consumed from each NOVA group. We then expressed the intake of MUPF and UPF as a percentage of the total daily grams consumed from all food items (g/d).

This methodological decision aligns with the core principles of the NOVA framework, which is currently endorsed and utilized by major international health organizations, including the Food and Agriculture Organization of the United Nations (FAO) [23], the United Nations Children’s Fund (UNICEF) [24], and the Pan American Health Organization (PAHO) [25]. These bodies emphasize a dietary priority of basing nutrition on minimally processed foods while avoiding ultra-processed products to prevent non-communicable diseases. Processed culinary ingredients (group 2) are rarely consumed alone and are inherently used to prepare MUPF, while processed foods (group 3) represent an intermediate category. By evaluating the two extremes of the food processing spectrum, we aimed to capture the most distinct and contrasting dietary patterns, providing a clearer epidemiological signal regarding the health impacts of industrial food processing on IMID risk.

We prioritized the weight-based proportion (% g/day) over energy-based metrics to capture the contribution of non-caloric or low-calorie ultra-processed items, such as artificially sweetened beverages and “light” products, reflecting the actual quantity of food consumed. To ensure the robustness of our findings, we also evaluated exposure using the percentage of total daily energy intake (% kcal/day), absolute energy intake (kcal/day), and absolute weight intake (g/day).

For statistical analysis, participants were categorized into tertiles based on the distribution of MUPF and UPF consumption in the study population.

### 2.4. Covariates

A comprehensive array of potential covariates was assessed at baseline through a detailed, self-administered questionnaire. The collected variables encompassed key sociodemographic factors, including sex (categorized as male or female), age (treated as a continuous variable in years), and the duration of university education (also continuous, in years). To account for lifestyle and behavioral influences, we evaluated smoking status, classifying participants as never, current, or former smokers. Physical activity was quantified in detail as the total metabolic equivalent of task (MET) minutes expended per week [26], handled as a continuous variable and additionally categorized into low (less than 500 MET-min/week) or high (500 MET-min/week or more) activity levels [27]. Sedentary behavior was captured through daily television watching time, classified as high (2 h or more per day) [28]. Sleep patterns were characterized by total nocturnal sleep duration, analyzed continuously in hours per day and categorically according to recommendations by the National Sleep Foundation as less than 6, 6 to 8, or more than 8 h [29], and siesta duration, recorded continuously in minutes per day and grouped as none, less than 30 min, or 30 min and above [30]. Anthropometric evaluation relied on previously validated self-reported weight and height [31] to calculate body mass index (BMI) as a continuous variable (kg/m^2^); for specific analyses, BMI was also classified into not overweight or overweight (≥25.0 kg/m^2^) [32]. Diet-related habits beyond the primary exposure included the evaluation of habitual supplement use, eating behaviors, and adherence to specific dietary regimens. Participants were asked whether they had habitually consumed vitamins and/or minerals (including calcium) during the previous year. Furthermore, we assessed the frequency of snacking between meals and whether participants followed a special diet (e.g., low-calorie, low-sodium, or medically prescribed diets) at baseline. Alcohol consumption (grams/day); total fiber intake (g/d); and the estimated total daily energy intake, a continuous variable measured in kilocalories, were also assessed. For women, pregnancy status at baseline was also recorded.

To address specific triggers for skin and immune-mediated conditions, additional adjustments were made for daily sun exposure, assessed as the average time spent sunbathing during summer and winter (h/d). Psychological distress, evaluated as baseline diagnosis of stress, anxiety, or depression, was incorporated as a potential trigger or amplifier of immune responses. Furthermore, total fiber intake, MedDiet pattern evaluated with the Mediterranean Diet Score (MDS) [33], and consumption of vegetables and fruits (g/d) was evaluated. Finally, information on prevalent health conditions was collected and used for the appropriate exclusion of participants from the analytic cohort to mitigate reverse causality.

### 2.5. Statistical Analysis

Time-to-event analysis was performed using Cox proportional hazard regression models to estimate hazard ratios (HRs) and 95% confidence intervals (CIs) for the association between MUPF or UPF consumption and the self-reported diagnosed incidence of IMIDs. Age was used as the underlying time variable. To ensure the robustness of the proportional hazard assumption, models were stratified by attained age categories (10-year intervals) and by follow-up time categories (5-year intervals), allowing the baseline hazard to vary across these strata. Two sequential multivariable models were constructed: Model 1 was adjusted for sex and age (both categorically, as a stratifying factor, and continually, as the underlying time variable). Model 2 included additional adjustments for years of university education, smoking status, alcohol consumption, BMI, following a special diet, snacking between meals, total physical activity, television viewing time, and nighttime sleep duration. The main exposure variables (% g/d of MUPF or UPF) were also adjusted for total daily energy intake using Willett’s residuals method to isolate the effect of dietary composition independently of total caloric intake. To account for changes in dietary habits over time, we performed a repeated measures analysis, updating dietary exposure data at the 10- and 20-year follow-ups using the same Cox regression approach. For intermediate periods or in the case of missing follow-up data, we employed the Last Observation Carried Forward (LOCF) method, ensuring that the models utilized the most recent dietary information available for each participant throughout their person-time at risk.

The proportional hazard assumption was tested for all models using Schoenfeld residual analysis; the nap duration variable violated this assumption and was consequently excluded from the final models, while all other covariates confirmed the validity of the model. To visually represent the adjusted cumulative self-reported diagnosed incidence across exposure groups, we constructed Nelson–Aalen cumulative hazard curves weighted by stabilized inverse probability weighting (IPW) derived from a multinomial logistic regression model. Furthermore, we conducted stratified analyses by sex, median age (34.6 years), years of follow-up, BMI (25 kg/m^2^), physical activity (500 MET-min/week), and smoking status to examine potential effect modifications, which were formally tested through multiplicative interaction terms and likelihood ratio tests.

To evaluate the robustness of our primary findings, we conducted an extensive series of sensitivity analyses. Beyond the main models, we performed additional adjustments for average sun exposure (summer and winter) and psychological distress (stress, anxiety, or depression). Furthermore, there were adjustments for total fiber (g/d), MedDiet adherence, and consumption of vegetables and fruits (g/d), specifically for the UPF models, to isolate the effect of food processing from overall dietary quality. These adjustments were not applied to MUPF models due to the inherent high correlation between minimally processed food consumption and these dietary quality markers, which would have led to significant overadjustment and multicollinearity. Furthermore, we included adjustments for macronutrient intake (carbohydrates, proteins, and fats) and a comprehensive set of micronutrients, including vitamins A, B1, B2, B3, B6, B12, C, D, and E; folic acid; and minerals such as calcium, iron, potassium, magnesium, zinc, phosphorus, and selenium, to account for nutritional density and potential mediation. The analysis was also extended to examine each specific IMID as an independent outcome to assess subtype-specific associations. Furthermore, we evaluated exposure using alternative metrics, including the percentage of total daily energy intake (% kcal/day), absolute UPF caloric intake (kcal/day), and absolute weight intake (g/day) for both MUPF and UPF. To mitigate potential bias and ensure the stability of our estimates, we performed successive exclusions of participants missing more than 30 items on the FFQ; individuals with prevalent dyslipidemia, hypertension, or depression at baseline; and those following a special diet. Additional exclusions were applied to users of dietary supplements and specific medications and pregnant women at baseline to account for temporary physiological immune modulation. Finally, we ensured that the overall association was not driven by a single condition by iteratively excluding each specific IMID subtype. All sensitivity analyses followed the same Cox regression approach used in the primary analysis.

All statistical analyses were performed using Stata software, version 17.0 (StataCorp LLC, College Station, TX, USA).

## 3. Results

### 3.1. Study Population and Follow-Up

**Figure 2:**
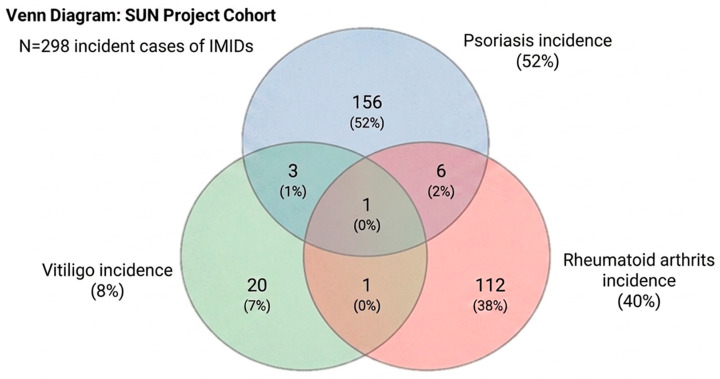
Venn diagram showing the overlap of incident cases of immune-mediated inflammatory diseases (IMIDs), psoriasis, rheumatoid arthritis, and vitiligo among participants during follow-up. The SUN Project. The Venn diagram illustrates the number and proportion of participants who developed new-onset (incident) psoriasis (*n* = 164, 55% of the total cases in the IMID cohort), rheumatoid arthritis (*n* = 119, 40%), and vitiligo (*n* = 24, 8%) over the study period. The numbers within each section represent the number of participants with that specific combination of diseases, with the corresponding percentage relative to the total cohort incident cases (*N* = 298). The diagram visualizes the co-occurrence of these autoimmune conditions, with a substantial number of cases presenting with only one disease (e.g., 156 participants had incident psoriasis alone) and a smaller subset developing more than one condition concurrently.

The final analytical sample comprised 15,874 participants after exclusions, contributing 227,324.05 person-years of follow-up (median follow-up: 15.1 years). A total of 298 incident IMID cases were identified, corresponding to a self-reported diagnosed incidence rate of 1.31 per 1000 person-years. Regarding the longitudinal dietary assessments, 10,264 participants (64.7%) completed the 10-year follow-up (C10) and 3287 participants (20.7%) completed the 20-year follow-up (C20), providing updated exposure data for the repeated measures analysis. The Venn diagram of IMID cases revealed that among the 298 cases, 156 (52.3%) had psoriasis only, 112 (37.6%) had rheumatoid arthritis only, 20 (6.7%) had vitiligo only, 6 (2.0%) had both psoriasis and rheumatoid arthritis, 3 (1.0%) had both psoriasis and vitiligo, and 1 (0.3%) had rheumatoid arthritis and vitiligo (Figure 2). The disease-specific self-reported diagnosed incidence rates were 0.73 per 1000 person-years for psoriasis, 0.52 for rheumatoid arthritis, and 0.11 for vitiligo.

### 3.2. Baseline Characteristics

Table 1 presents the baseline characteristics of the 15,874 participants. The mean age was 36.6 years (SD = 11.3), 62.0% were female, and the average university education was 5.1 years. Regarding health and lifestyle, 68.0% were not overweight (BMI < 25 kg/m^2^), 68.5% reported physical activity levels ≥ 500 METs-min/week, and 50.3% were never smokers. Prevalent conditions included dyslipidemia (15.6%), depression (10.7%), and hypertension (8.5%). The mean dietary weight was composed of 71.9% MUPF (ranging from 59.2% in T1 to 83.4% in T3) and 12.7% UPF (ranging from 5.3% in T1 to 21.8% in T3). Participants in the highest tertile of MUPF consumption (T3) were more likely to be female (78.5% vs. 43.7% in T1), not overweight (72.8% vs. 62.5%), and never smokers (54.8% vs. 44.6%). They also reported higher physical activity (71.4%), more frequent use of dietary supplements (22.7%), and a higher prevalence of following special diets (10.7% vs. 5.0%). In contrast, those in the highest tertile of UPF consumption (T3) were significantly younger (32.9 vs. 41.0 years in T1) and more likely to be male (44.7% vs. 32.0%). These participants reported less healthy behaviors, including lower physical activity (64.3% vs. 72.6% in T1), a higher frequency of snacking between meals (42.6% vs. 25.9%), and a higher prevalence of long sleep duration (15.5% vs. 11.3%). Notably, high UPF consumers had a lower prevalence of baseline hypertension (6.6% vs. 10.9%) and dyslipidemia (12.2% vs. 20.0%), likely reflecting their younger age profile.

The nutritional profile of the cohort varied across the spectrum of food processing (Supplementary Table S2). Participants in the highest tertile (T3) of UPF consumption showed a higher intake of sodium, saturated fats, and trans fats compared to those in the lowest tertile (T1). Conversely, high UPF intake was characterized by a marked reduction in total dietary fiber and vegetal protein. Regarding micronutrients, a high-UPF pattern was associated with a generalized depletion of essential elements for immune homeostasis. Specifically, T3 participants showed lower intakes of vitamin A, vitamin C, folic acid, potassium, magnesium, zinc, and vitamin D. In contrast, the MUPF pattern exhibited a superior nutritional density. Participants in the highest MUPF tertile had significantly higher intakes of fiber, vitamin A, and vitamin C.

### 3.3. Main Associations

Table 2 shows the association between MUPF and UPF consumption and the risk of specific diseases and the exploratory composite outcome (IMIDs). Regarding specific diseases, higher UPF consumption significantly increased the risk of both psoriasis (HR = 1.63, 95% CI: 1.08–2.45; *p*-trend = 0.018) and rheumatoid arthritis (HR = 1.97, 95% CI: 1.19–3.26; *p*-trend = 0.006). Conversely, higher MUPF consumption was significantly associated with a lower risk of psoriasis (T3 vs. T1: HR = 0.60, 95% CI: 0.40–0.92; *p*-trend = 0.014), while the inverse association for rheumatoid arthritis did not reach statistical significance (HR = 0.71, 95% CI: 0.44–1.16). Consistent with these findings, the exploratory composite IMID endpoint showed that higher UPF consumption was positively associated with risk. Compared to T1 (<8.2%), participants in T3 (>14.4%) had an HR of 1.80 (95% CI: 1.31–2.45; *p*-trend < 0.001). In the repeated measures model, this association remained significant (T3 vs. T1: HR = 1.59, 95% CI: 1.15–2.20). Similarly, higher MUPF intake was significantly associated with a reduced risk of the exploratory IMID outcome (T3 vs. T1: HR = 0.64, 95% CI: 0.47–0.87; *p*-trend = 0.003), with consistent results in the repeated measures analyses over 20 years of follow-up (T3 vs. T1: HR = 0.72, 95% CI: 0.52–0.99). No significant associations were found for vitiligo individually.

**Figure 3:**
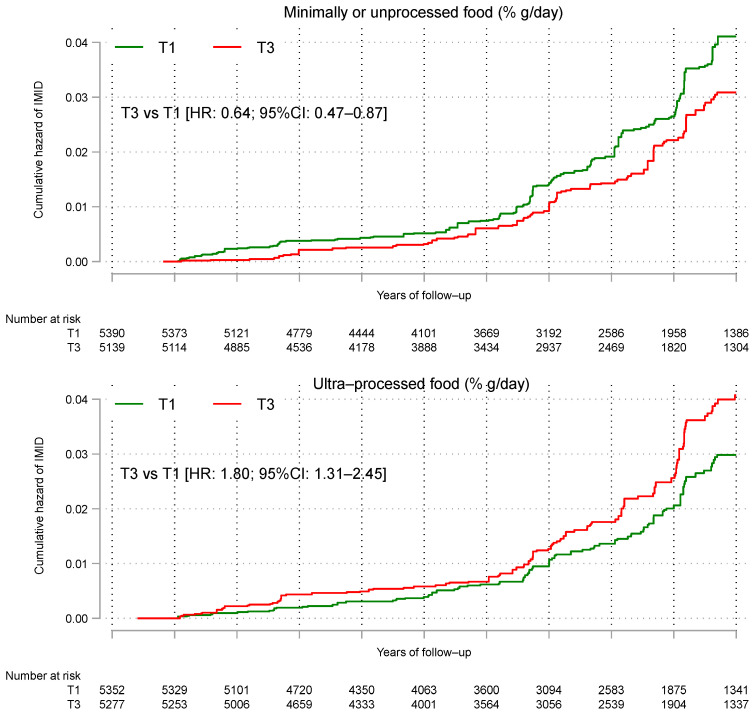
Nelson–Aalen curves of cumulative risk of immune-mediated inflammatory diseases (IMIDs) according to intake groups of minimally processed or unprocessed foods (MUPFs) and ultra-processed foods (UPFs) over the follow-up period. The SUN Project. Nelson–Aalen cumulative hazard curves for IMIDs are presented according to tertiles of minimally or unprocessed food intake (**upper panel**) and ultra-processed food intake (**lower panel**). For enhanced visual clarity and comparison of the extremes of consumption, only the lowest (T1) and highest (T3) tertiles are displayed, with the intermediate tertile (T2) omitted from the graphical representation. The number at risk at baseline and at 2-year follow-up intervals is shown below the graphs. All models are adjusted for sex, university education, smoking status, body mass index, following a special diet, snacking between meals, total physical activity, television viewing time, nighttime sleep duration, alcohol consumption, and total daily energy intake using inverse probability weighting. Follow-up time is presented in years. T1 and T3 refer to intake tertiles based on the percentage of grams per day.

These divergent risk patterns according to the degree of food processing are illustrated in the Nelson–Aalen cumulative hazard curves (Figure 3), which demonstrate a clear separation in cumulative self-reported diagnosed incidence over the 20-year follow-up period.

### 3.4. Modified Effect and Sensitivity Analysis

**Figure 4:**
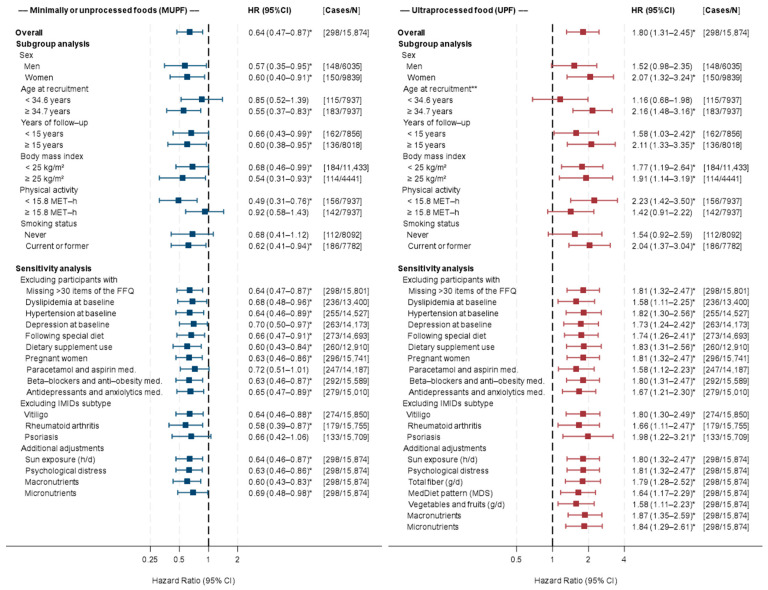
Forest plot of stratified subgroup analyses and sensitivity analyses for the association between consumption of minimally or unprocessed foods (MUPFs) and ultra-processed foods (UPFs) and the risk of immune-mediated inflammatory diseases (IMIDs). The SUN Project. Hazard ratios (HRs) and 95% confidence intervals (CIs) for the association between higher consumption of MUPFs (blue squares) and UPFs (red squares) (the highest tertile, T3, vs. the lowest tertile, T1) and IMID risk, stratified by potential effect modifiers. For each subgroup, the square represents the point estimate (HR), the horizontal line represents the 95% CI, and the number of incident cases and total person-years of follow-up are provided in brackets. An single asterisk (*) denotes a statistically significant HR (*p* < 0.05). A double asterisk (**) denotes a statistically significant *p* for interaction (*p* < 0.05) for that specific subgroup variable. All models are adjusted for sex, college education, smoking status, body mass index, following a special diet, snacking between meals, total physical activity, time watching television, duration of nighttime sleep, alcohol consumption, and total daily energy intake (through the residuals method). Additional adjustments were made for daily sun exposure (h/d), psychological distress (baseline diagnosis of stress, anxiety, or depression), macronutrients (carbohydrates, proteins, and fats), and micronutrients (vitamins A, B1, B2, B3, B6, B12, C, D, and E; folic acid; calcium; iron; potassium; magnesium; zinc; phosphorus; and selenium). Furthermore, total fiber intake, MedDiet pattern (MDS 0–9 points), and consumption of vegetables and fruits (g/d) were evaluated specifically for the UPF models to isolate the effect of food processing from overall dietary quality. These adjustments were not applied to MUPF models due to the inherent high correlation between minimally processed food consumption and these dietary quality markers, which would have led to significant overadjustment and multicollinearity. The *x*-axis (hazard ratio) is plotted on a logarithmic scale.

Subgroup analyses demonstrated that the inverse association between higher MUPF consumption and reduced IMID risk was remarkably consistent across most population strata (Figure 4, Supplementary Figure S1). Notably, the inverse association was more pronounced among participants with a BMI < 25 kg/m^2^ (HR = 0.68, 95% CI: 0.46–0.99) and those with higher physical activity levels (≥15.8 MET-h/week: HR = 0.49, 95% CI: 0.31–0.76).

For UPF consumption, a significant interaction was observed with age (*p*-interaction < 0.05). The harmful association was substantially stronger among older participants (≥34.7 years: HR = 2.16, 95% CI: 1.48–3.16) compared to younger participants (<34.6 years: HR = 1.16, 95% CI: 0.68–1.98). Furthermore, the associations for both MUPF and UPF remained robust across all sensitivity scenarios, persisting even after additional adjustments for sun exposure, psychological distress, and overall dietary quality markers. The results were likewise consistent in models adjusted for macronutrients and a comprehensive panel of immunomodulatory micronutrients. Additionally, the associations persisted after excluding participants with baseline dyslipidemia, hypertension, or depression, as well as pregnant women and users of paracetamol or aspirin (Figure 4, Supplementary Figures S1 and S2).

When the exposure was modeled as the energy-adjusted proportion of UPF consumption (% of total kcal/day) instead of the gram-based proportion, the positive association with the composite exploratory IMID risk remained statistically significant in the multivariable-adjusted model, though with a lower magnitude (T3 vs. T1: HR = 1.35, 95% CI: 1.01–1.81; *p*-trend = 0.045). However, it is important to note that when using this energy-based metric, the associations for the individual diseases (psoriasis, rheumatoid arthritis, and vitiligo) did not reach formal statistical significance (Supplementary Table S3). Conversely, a higher percentage of energy from MUPF was strongly associated with a lower risk of IMIDs (T3 vs. T1: HR = 0.68, 95% CI: 0.50–0.92; *p*-trend = 0.014) (Supplementary Table S3). When considering the absolute energy intake from UPF (kcal/day), the association with the composite outcome lost statistical significance in the fully adjusted model (HR = 1.16, 95% CI: 0.81–1.67; *p*-trend = 0.387). However, absolute energy intake from MUPF maintained a significant protective association with IMID risk (T3 vs. T1: HR = 0.72, 95% CI: 0.53–0.97; *p*-trend = 0.029) (Supplementary Table S4). In models using absolute weight intake (g/day), higher MUPF consumption maintained a significant inverse association with IMID risk (T3 vs. T1: HR = 0.61, 95% CI: 0.45–0.83; *p*-trend = 0.001). For UPF, while the absolute weight intake was positively associated with rheumatoid arthritis (T3 vs. T1: HR = 1.59, 95% CI: 0.98–2.58; *p*-trend = 0.048), the association for total IMID risk did not reach formal significance in the multivariable absolute weight intake (g/day) model (HR = 1.29, 95% CI: 0.95–1.75; *p*-trend = 0.092) (Supplementary Table S5).

## 4. Discussion

This prospective cohort study provides novel evidence that the degree of food processing is significantly associated with the long-term risk of developing IMIDs. Our findings reveal a clear divergence in risk; a dietary pattern higher in MUPF was associated with a lower self-reported diagnosed incidence of IMIDs, whereas a pattern higher in UPF was associated with a high risk. These results contribute to the evolving understanding that diet may act as a pivotal modifiable environmental factor in association with these conditions.

Our observations align with and extend the existing literature on diet and immune-mediated conditions [10,11,12,13,34,35]. The inverse association observed for higher MUPF consumption resonates with the benefits of whole-food dietary patterns. Such diets are inherently rich in anti-inflammatory and antioxidant compounds, which may help modulate immune function [35]. Conversely, the association between UPF intake and IMID risk aligns with research on the health impacts of food processing [36], suggesting that ultra-processed patterns may correlate with environments favoring disease development, though the exact biological pathways require further investigation.

The disease-specific analyses from our study offer further nuance. While both psoriasis and rheumatoid arthritis showed sensitivity to UPF intake, similar to other studies [37,38], the protective association with MUPF was specifically observed for psoriasis. This finding is notable given that Mendelian randomization studies have already established a causal link between higher BMI, often related to dietary patterns, and psoriasis risk [39]. However, it is important to emphasize that, as part of an observational study investigating disease incidence, our findings are not directly comparable to intervention studies that examine the effects of dietary patterns on the severity or clinical course of existing IMIDs.

The non-significant results for vitiligo should be interpreted with caution. While these findings might be attributed to the limited statistical power from a smaller number of incident cases (*n* = 24), the possibility that there is no genuine association between UPF consumption and the development of this specific condition cannot be excluded. From a pathophysiological standpoint, psoriasis and rheumatoid arthritis are more closely linked to systemic metabolic alterations and adiposity, whereas vitiligo typically presents with a more localized immune profile and a lower degree of metabolic comorbidity. This heterogeneity suggests that the influence of diet may be condition-specific, modulated by distinct genetic and pathophysiological backgrounds [40].

The biological plausibility of these associations can be grounded in several interconnected mechanisms. Diets rich in MUPFs typically provide a high density of nutrients and fiber that support gut barrier integrity and promote a healthy gut microbiota [4,10]. In contrast, industrial processes used to manufacture UPFs often dismantle the natural architecture of foods and introduces additives, such as emulsifiers and artificial sweeteners, that may disrupt the delicate balance of the gut microbiome and compromise intestinal barrier function [23,41,42,43]. This disruption can potentially trigger abnormal immune responses [10]. Additionally, specific dietary components, common in UPFs, such as saturated fats, can activate pro-inflammatory pathways in innate immune cells [4,10].

Our stratified analyses offer deeper insights. A significant effect modification by age was observed for UPF consumption, where the harmful association was substantially stronger among older participants, possibly reflecting age-related dysregulation of the immune system [44,45]. Additionally, the protective association of MUPFs was statistically significant specifically among current or former smokers, suggesting that the antioxidant properties of MUPFs may help counteract the oxidative stress induced by smoking [46,47].

An interesting finding was the attenuation of the UPF-IMID association when the energy-adjusted proportion was modeled instead of the absolute gram-based proportion. Although the sensitivity of the results to the exposure metric could be viewed as a methodological limitation, this divergence suggests that the association between UPF and health outcomes may be related to factors beyond caloric density. A large prospective study on systemic lupus erythematosus similarly found that UPF intake measured in servings/day and grams/day showed stronger associations with disease risk than the percentage of total caloric intake [12]. This divergence suggests that many non-caloric components of UPFs, such as additives (emulsifiers, artificial colors, and non-caloric sweeteners), contaminants from packaging, and advanced glycation end products, may play a role in the observed associations [42,48]. With over 82% of UPFs containing at least one cosmetic additive and nearly 25% containing six or more [49], a weight-based metric may better reflect the total burden of these non-nutritive, potentially pro-inflammatory exposures than caloric density. Conversely, energy-based metrics may dilute these associations by grouping low-calorie, highly processed items (e.g., diet sodas and processed meats with additives) with energy-dense, minimally processed foods (e.g., extra virgin olive oil or nuts).

Regarding the study’s limitations, the use of a composite primary endpoint, while grounded in shared inflammatory pathways, primarily reflects the incidence of psoriasis and rheumatoid arthritis, which comprised the majority of cases. Consequently, less common IMIDs not captured by the baseline questionnaire were not evaluated. However, the consistent results observed in individual analyses for psoriasis and rheumatoid arthritis support the robustness of the overall association.

Additionally, outcome ascertainment relied on self-reported medical diagnoses, introducing potential misclassification bias. Although self-reported IMIDs have not been specifically validated within the SUN Project, the cohort has demonstrated high diagnostic validity for other conditions, such as components of metabolic syndrome [17,50], depression [51], and gastric or duodenal ulcers [52]. While the significant quality-of-life impact of these diseases often leads to formal medical consultation, we cannot exclude detection bias; thus, our results likely reflect diagnosed incidence rather than true biological incidence. This limitation is compounded by a lack of detailed clinical phenotyping, as these biologically heterogeneous diseases may be influenced by diet through distinct pathways not captured by our broad categories. Furthermore, due to data constraints, we could not stratify by disease severity, serological status, or specific subtypes (e.g., segmental versus non-segmental vitiligo), nor could we exclude diagnostic misclassification in cases where psoriasis and rheumatoid arthritis overlapped, which may potentially represent psoriatic arthritis. Regarding vitiligo, the analysis was likely underpowered to detect significant associations; consequently, these results remain inconclusive and require further evaluation.

Finally, the use of a self-administered FFQ may introduce recall and social desirability biases, potentially leading to the under-reporting of UPFs or over-reporting of healthy foods. However, the FFQ used has been previously validated within the SUN cohort, demonstrating acceptable validity and reproducibility [19]. Nonetheless, because the FFQ was originally designed to assess nutrient intake rather than industrial processing, the post hoc application of the NOVA classification may have resulted in some exposure misclassification, a common constraint in legacy cohorts.

Despite these limitations, the high educational level of our participants, many of whom are health professionals, minimizes reporting errors and enhances the internal validity of both dietary and diagnostic data [14,15,16]. While the prospective design helps minimize reverse causality, the observational nature of this research means that residual confounding cannot be entirely excluded. UPF consumption might serve as a marker for other sub-optimal health behaviors; however, the persistence of the association after adjusting for overall diet quality and specific nutrients suggests that food processing itself may be independently relevant. Ultimately, these results demonstrate plausible longitudinal associations rather than definitive proof of causation, and future experimental data are needed to establish a causal link.

The strengths of this study lend considerable weight to its findings. These include the prospective design, which minimizes reverse causality; the large sample size and extended median follow-up of over 15 years; and the ability to adjust for a wide array of potential confounders, including detailed lifestyle and clinical factors. This study is among the first prospective investigations to examine the association between the consumption of both UPF and MUPF, defined by the NOVA classification, and the risk of multiple, specific IMIDs. While recent prospective work has linked UPF intake to the risk of individual autoimmune conditions like systemic lupus erythematosus [12], the dual assessment of UPF and MUPF in relation to a combined autoimmune outcome and specific diseases like psoriasis, rheumatoid arthritis, and vitiligo remains novel.

## 5. Conclusions

The findings of this study suggest that a diet centered on MUPF is associated with a lower risk of self-reported diagnosed incidence of psoriasis, while a diet high in UPF is associated with a significantly higher risk of both self-reported diagnosed psoriasis and rheumatoid arthritis. These findings underscore the importance of looking beyond nutrient-based guidelines to consider the holistic quality and processing of food in dietary recommendations. Further research, particularly interventional studies, is needed to establish causality and disentangle the specific components within UPF that may influence IMIDs.

## Figures and Tables

**Table 1:** Baseline characteristics of participants according to extreme tertiles of minimally or unprocessed food (MUPF) and ultra-processed food (UPF) consumption. The SUN Project.

Characteristics	Total	Extreme Tertiles of Percentage of MUPF Gram Consumption			Extreme Tertiles of Percentage of UPF Gram Consumption		
		T1 (<68.4%)	T3 (>77.7%)	*p*-Trend	T1 (<8.2%)	T3 (>14.4%)	*p*-Trend
N	15,874	5292	5291		5292	5291	
Sociodemographic							
Age, years	36.62 ± 11.27	36.70 ± 11.08	37.07 ± 11.68	0.092	41.03 ± 11.87	32.86 ± 9.43	**<0.001**
Sex, female	9839 (62.0%)	2315 (43.7%)	4156 (78.5%)	**<0.001**	3596 (68.0%)	2927 (55.3%)	**<0.001**
Years of university education	5.06 ± 1.50	5.18 ± 1.54	4.93 ± 1.45	**<0.001**	5.11 ± 1.54	5.04 ± 1.48	**0.016**
Health and lifestyle							
Not overweight (BMI < 25)	10,788 (68.0%)	3309 (62.5%)	3854 (72.8%)	**<0.001**	3592 (67.9%)	3594 (67.9%)	0.956
Physical activity, ≥500 METs min/wk	10,873 (68.5%)	3514 (66.4%)	3777 (71.4%)	**<0.001**	3842 (72.6%)	3400 (64.3%)	**<0.001**
Smoking, never smoked	7991 (50.3%)	2361 (44.6%)	2899 (54.8%)	**<0.001**	2490 (47.1%)	2809 (53.1%)	**<0.001**
Hypertension diagnosis	1347 (8.5%)	533 (10.1%)	414 (7.8%)	**<0.001**	577 (10.9%)	348 (6.6%)	**<0.001**
Dyslipidemia	2474 (15.6%)	869 (16.4%)	815 (15.4%)	0.153	1058 (20.0%)	646 (12.2%)	**<0.001**
Depression	1701 (10.7%)	545 (10.3%)	632 (11.9%)	**0.007**	643 (12.2%)	509 (9.6%)	**<0.001**
Paracetamol and aspirin use	1687 (10.6%)	549 (10.4%)	548 (10.4%)	0.977	565 (10.7%)	574 (10.8%)	0.775
Beta-blockers/anti-obesity med.	285 (1.8%)	97 (1.8%)	117 (2.2%)	0.167	125 (2.4%)	79 (1.5%)	**0.001**
Antidepressants/anxiolytics use	864 (5.4%)	316 (6.0%)	280 (5.3%)	0.130	304 (5.7%)	289 (5.5%)	0.528
Dietary intake							
Total MUPF (g/day)	1672.34 ± 4.61	1214.76 ± 4.53	2189.34 ± 7.74	**<0.001**	2066.07 ± 8.44	1293.97 ± 5.46	**<0.001**
Total UPF (g/day)	279.62 ± 1.39	378.46 ± 3.10	188.52 ± 1.23	**<0.001**	146.89 ± 0.86	441.72 ± 2.72	**<0.001**
Total energy intake (kcal/day)	2351.19 ± 611.78	2372.33 ± 631.62	2329.89 ± 604.24	**<0.001**	2228.60 ± 599.05	2258.50 ± 531.62	**<0.001**
Following a special diet	1181 (7.4%)	266 (5.0%)	565 (10.7%)	**<0.001**	564 (10.7%)	269 (5.1%)	**<0.001**
Snacking between meals	5458 (34.4%)	1935 (36.6%)	1678 (31.7%)	**<0.001**	1369 (25.9%)	2256 (42.6%)	**<0.001**
Supplement use	2964 (19.5%)	856 (16.9%)	1147 (22.7%)	**<0.001**	1026 (20.4%)	953 (18.7%)	**0.037**
Sleep characteristics							
Short sleep duration (<6 h/d)	818 (5.2%)	259 (4.9%)	307 (5.8%)	0.241	288 (5.4%)	243 (4.6%)	**<0.001**
Long sleep duration (>8 h/d)	2143 (13.5%)	713 (13.5%)	712 (13.5%)	0.241	598 (11.3%)	822 (15.5%)	**<0.001**
Daytime napping (30–60 min/day)	2238 (14.1%)	773 (14.6%)	711 (13.4%)	**0.039**	761 (14.4%)	742 (14.0%)	0.301
Daytime napping (>60 min/day)	815 (5.1%)	295 (5.6%)	273 (5.2%)	**0.039**	253 (4.8%)	314 (5.9%)	0.301

Values are presented as means ± standard deviations for continuous variables and as frequencies n (%) for categorical variables. G1 refers to the group with predominant consumption of minimally or unprocessed foods, while G4 refers to the group with predominant consumption of ultra-processed foods. T1 and T3 represent, respectively, the first and third tertiles of consumption within each group. The *p*-trend was calculated using linear regression models (continuous variables) or logistic regression (categorical variables) comparing the extreme tertiles (T1 vs. T3). Values of *p* < 0.05 are highlighted in bold. Abbreviations: MET: metabolic equivalents; UPF: ultra-processed foods.

**Table 2:** Hazard ratios (95% CIs) for the association between the proportion of total weight intake (% of grams/day) from minimally processed foods (MUPFs) and ultra-processed foods (UPFs) and the self-reported diagnosed incidence of immune-mediated inflammatory diseases. The SUN Project.

	Tertiles of Percentage of MUPF Gram Consumption				Tertiles of Percentage of UPF Gram Consumption			
	T1 (<68.4%)	T2 (68.4–77.7%)	T3 (>77.7%)	*p*-Trend	T1 (<8.2%)	T2 (8.2–14.4%)	T3 (>14.4%)	*p*-Trend
Immune-mediated inflammatory diseases								
Persons-years	77,174	75,862	74,288		76,093	75,854	75,377	
Cases/N	134/5292	79/5291	85/5291		90/5292	103/5291	105/5291	
Unadjusted	Ref	0.62 (0.47–0.82)	0.65 (0.49–0.85)	**0.001**	Ref	1.50 (1.12–1.99)	1.88 (1.40–2.53)	**<0.001**
Age and sex adjusted	Ref	0.62 (0.46–0.82)	0.61 (0.46–0.82)	**0.001**	Ref	1.58 (1.18–2.11)	1.95 (1.44–2.65)	**<0.001**
Multivariable adjusted	Ref	0.63 (0.47–0.84)	0.64 (0.47–0.87)	**0.003**	Ref	1.49 (1.11–2.00)	1.80 (1.31–2.45)	**<0.001**
Repeated measurements	Ref	0.77 (0.57–1.03)	0.72 (0.52–0.99)	**0.039**	Ref	1.52 (1.13–2.05)	1.59 (1.15–2.20)	**0.005**
Psoriasis								
Cases/N	79/5292	43/5291	43/5291		48/5292	50/5291	67/5291	
Unadjusted	Ref	0.56 (0.39–0.82)	0.56 (0.39–0.82)	**0.001**	Ref	1.18 (0.79–1.76)	1.76 (1.20–2.60)	**0.004**
Age and sex adjusted	Ref	0.59 (0.40–0.86)	0.58 (0.39–0.86)	**0.004**	Ref	1.22 (0.81–1.82)	1.77 (1.19–2.64)	**0.004**
Multivariable adjusted	Ref	0.60 (0.41–0.89)	0.60 (0.40–0.92)	**0.014**	Ref	1.16 (0.77–1.75)	1.63 (1.08–2.45)	**0.018**
Repeated measurements	Ref	0.75 (0.51–1.10)	0.61 (0.39–0.95)	**0.025**	Ref	1.31 (0.86–1.98)	1.56 (1.02–2.39)	**0.043**
Rheumatoid arthritis								
Cases/N	49/5292	31/5291	39/5291		40/5292	45/5291	34/5291	
Unadjusted	Ref	0.69 (0.44–1.08)	0.79 (0.52–1.20)	0.255	Ref	1.75 (1.13–2.69)	1.89 (1.17–3.05)	**0.006**
Age and sex adjusted	Ref	0.64 (0.41–1.02)	0.68 (0.43–1.07)	0.087	Ref	1.94 (1.25–3.00)	2.10 (1.29–3.44)	**0.002**
Multivariable adjusted	Ref	0.66 (0.41–1.06)	0.71 (0.44–1.16)	0.175	Ref	1.82 (1.16–2.84)	1.97 (1.19–3.26)	**0.006**
Repeated measurements	Ref	0.72 (0.44–1.19)	0.92 (0.56–1.52)	0.750	Ref	1.88 (1.19–2.98)	1.87 (1.10–3.18)	**0.013**
Vitiligo								
Cases/N	9/5292	8/5291	7/5291		6/5292	10/5291	8/5291	
Unadjusted	Ref	0.93 (0.36–2.41)	0.78 (0.29–2.10)	0.626	Ref	2.23 (0.80–6.22)	2.24 (0.74–6.76)	0.147
Age and sex adjusted	Ref	0.83 (0.31–2.18)	0.66 (0.23–1.88)	0.436	Ref	2.19 (0.78–6.21)	2.18 (0.70–6.80)	0.177
Multivariable adjusted	Ref	0.72 (0.27–1.96)	0.63 (0.21–1.90)	0.411	Ref	1.88 (0.66–5.36)	1.66 (0.52–5.33)	0.409

Hazard ratios (HRs) and 95% confidence intervals (CIs) compare upper tertiles (T2–T3) vs. the lowest tertile (T1) of dietary consumption. All models are adjusted for sex, college education, smoking status, body mass index, following a special diet, snacking between meals, total physical activity, time watching television, duration of nighttime sleep, alcohol consumption, and total daily energy intake (through the residuals method). For the repeated measures models, dietary exposure data were dynamically updated for the *n* = 10,264 (64.7%) participants who completed the 10-year follow-up and the *n* = 3287 (20.7%) participants who completed the 20-year follow-up. For vitiligo, only baseline dietary data were analyzed; models using repeated measures were not performed due to the low number of incident cases, which limited statistical power and prevented model convergence. Values of *p* < 0.05 are highlighted in bold. Abbreviations: HR, hazard ratio; CI, confidence interval.

## Data Availability

The datasets supporting the findings of the SUN Project are available upon reasonable request. Interested researchers may contact the Department of Preventive Medicine and Public Health at the University of Navarra (Pamplona, Spain) by emailing sun@unav.es for information regarding data access.

## Supplementary Materials

The following supporting information can be downloaded at https://www.mdpi.com/article/10.3390/nu18121969/s1: Supplementary Table S1. Classification of food items into minimally processed or unprocessed foods (MUPFs) and ultra-processed foods (UPFs) according to the NOVA food processing system (The SUN Project, Navarra, Spain). Supplementary Table S2. Nutritional profile and food group intake across extreme tertiles of adherence to minimally or unprocessed food (MUPF) and ultra-processed food (UPF). Supplementary Table S3. Hazard ratios (95% CIs) for the association between the relative energy intake from minimally or unprocessed foods (MUPFs) and ultra-processed foods (UPFs) (% of kcal/day) and the self-reported diagnosed incidence of immune-mediated inflammatory diseases (IMIDs). SUN Project. Supplementary Table S4. Hazard ratios (95% CIs) for the association between the absolute energy intake of minimally or unprocessed foods (MUPFs) and ultra-processed foods (UPFs) (kcal/day) with the self-reported diagnosed incidence of immune-mediated inflammatory diseases (IMIDs). The SUN Project. Supplementary Table S5. Hazard ratios (95% CIs) for the association between the absolute weight intake (grams/day) of minimally processed foods (MUPFs) and ultra-processed foods (UPFs) and the self-reported diagnosed incidence of immune-mediated inflammatory diseases (IMIDs). The SUN Project. Supplementary Figure S1. Forest plot of stratified subgroup analyses and sensitivity analyses for the association between consumption of minimally or unprocessed foods (MUPFs) and the risk of immune-mediated inflammatory diseases (IMIDs). The SUN Project. Supplementary Figure S2. Forest plot of stratified subgroup analyses and sensitivity analyses for the association between consumption of ultra-processed foods (UPFs) and the risk of immune-mediated inflammatory diseases (IMIDs). The SUN Project.

## Abbreviations

The following abbreviations are used in this manuscript.

BMI	Body mass index
CI	Confidence interval
FFQ	Food frequency questionnaire
HR	Hazard ratio
IMIDs	Immune-mediated inflammatory diseases
MDS	Mediterranean Diet Score
MET	Metabolic equivalent of task
MUPF	Minimally or unprocessed food
SLE	Systemic lupus erythematosus
SUN	Seguimiento Universidad de Navarra
UPF	Ultra-processed food
