# Supplementary material for: Degree of Food Processing and the Risk of Immune-Mediated Inflammatory Diseases: A Prospective Analysis of the SUN Cohort
Source: Nutrients. 2026 Jun 18;18(12):1969. doi: 10.3390/nu18121969 (PMC13304887; doi:10.3390/nu18121969)
Supplement: Supplementary file 1 [file nutrients-18-01969-s001.zip › nutrients-4240516-supplementary.pdf]

Degree of food processing and the risk of immune-mediated inflammatory diseases: A prospective analysis of the SUN Cohort

Supplementary Table S1. Classification of food items in minimally or unprocessed food (MUPF) and ultra-processed food (UPF) according to the NOVA food processing system. The SUN Project.

NOVA Group	Definition	FFQ items
1) Unprocessed or minimally processed foods	Edible parts of plants or animals, or foods obtained directly from nature, are altered only by minimal processes such as cleaning, grinding, refrigeration, freezing, pasteurization, or cooking.	Cereals: White rice, pasta (noodles, macaroni, spaghetti). Milk/Dairy: Whole milk, semi-skimmed milk, skimmed milk, yogurt, skimmed yogurt, curd. Meat: Chicken with/without skin, veal, pork, lamb, rabbit, liver and other viscera, bacon, pancetta. Eggs: Hen eggs. Fish: White and blue fish, cod, oysters, clams, mussels, shrimps, squid, octopus, cuttlefish. Vegetables: Chard, spinach, cabbage, cauliflower, broccoli, lettuce, endives, escarole, tomato, carrot, pumpkin, green bean, eggplant, zucchini, cucumber, pepper, asparagus, gazpacho, borage, thistle, baked/boiled potatoes. Fruits: Orange, grapefruit, tangerine, banana, apple, pear, strawberry, peach, apricot, nectarine, cherries, plum, fig, watermelon, melon, grape, dates, dried figs, raisins, prunes, avocado, mango, papaya, kiwi. Legumes: Lentils, chickpeas, beans, peas. Nuts: Almonds, peanuts, walnuts, hazelnuts. Non-alcoholic beverages: Natural orange juice, other natural juices, caffeinated/decaffeinated coffee, water.
2) Processed culinary ingredients	Substances obtained directly from group 1 foods or from nature by processes such as pressing, refining, crushing, milling, and drying.	Oils/Fats: Olive oil, sunflower oil, corn oil, lard, other oils, butter. Sugars: Sugar. Condiments: Tabasco, pepper, salt, honey.
3) Processed foods	Relatively simple products made by adding sugar, oil, salt, or other group 2 substances to group 1 foods.	Cereals: White bread, wholegrain bread. Milk/Dairy: Condensed milk, cream, cheese in portions, cured and semi-cured cheeses, fresh cheese. Meat: Ham cured in salt. Fish: Dried salted and smoked fish. Fruits: Fruits in syrup, fruits in natural juice, jam, olives. Homemade: Homemade pastries. Sauces: Tomato sauce. Alcoholic beverages: Red wine, other wines, beer.
Ultra-processed foods	Industrial formulations made mostly or entirely from substances derived from foods and additives, with little or no whole food, are designed to be convenient, highly palatable, and ready-to-consume.	Cereals: Breakfast cereals. Milk/Dairy: Milkshakes, petit suisse (sweetened cheese), custard, ice cream. Meat: Boiled ham, chorizo, salchichon, mortadella, sausages, pâté, foie-gras, blood sausage, hamburgers, sobrasada, meatballs. Pre-cooked: Pre-cooked pizza, croquettes, patties, instant soups and creams. Sugars/Sweets: María cookies, chocolate cookies, cupcakes, doughnuts, fritters, croissants, cakes, churros, chocolate, nougat, marzipan, tea pastries. Oils/Fats: Margarine. Salty snacks: Potato chips. Sauces: Mayonnaise. Non-alcoholic beverages: Soft drinks/sodas (regular and light), packaged juices. Alcoholic beverages: Spirits (whiskey, gin, cognac, anisette). Other: Artificial sweeteners.

Items were categorized by two independent dietitians following standardized criteria. Discrepancies were resolved by consensus or a third researcher. Composite items or those with unspecified preparation were classified by their primary ingredient or the most common consumption pattern in Spain. In cases of ambiguity, a conservative approach was adopted, assigning the item to the lowest possible processing level or the most typically consumed item in Spain. While all NOVA groups are presented for transparency, only Group 1 (MUPF) and Group 4 (UPF) were used as primary exposure endpoints in the statistical analyses.

Degree of food processing and the risk of immune-mediated inflammatory diseases: A prospective analysis of the SUN Cohort

Supplementary Table S2. Nutritional profile and food group intake across extreme tertiles of adherence to the minimally or unprocessed food (MUPF) and the ultraprocessed food (UPF).

Variables	Total	Tertiles of percentual of MUPF grams consumption		Tertiles of percentual of UPF grams consumption	
		T1 (<68.4%)	T3 (>77.7%)	T1 (<8.2%)	T3 (>14.4%)
Energy intake (kcal/day)	2351.2 ± 611.8	2485.0 ± 631.2	2204.6 ± 585.8	2228.6 ± 599.0	2458.5 ± 631.6
Macronutrients					
Carbohydrate intake (% kcal/d)	43.4 ± 7.3	42.5 ± 7.2	44.6 ± 7.6	44.3 ± 8.0	43.1 ± 6.9
Protein intake (% kcal/d)	18.2 ± 3.2	16.8 ± 2.8	19.7 ± 3.4	19.1 ± 3.5	17.2 ± 3.0
Animal protein (% kcal/d)	12.9 ± 3.4	11.9 ± 3.1	13.9 ± 3.8	13.2 ± 3.8	12.3 ± 3.2
Vegetal protein (% kcal/d)	5.4 ± 1.3	5.0 ± 1.1	5.9 ± 1.4	6.0 ± 1.4	4.9 ± 1.1
Fat intake (% kcal/d)	36.4 ± 6.5	37.4 ± 6.5	34.8 ± 6.6	34.3 ± 6.9	38.0 ± 6.1
SFA (% kcal/d)	12.5 ± 3.2	13.2 ± 3.1	11.6 ± 3.2	11.2 ± 3.1	13.6 ± 3.0
MUFA (% kcal/d)	15.8 ± 3.7	16.2 ± 3.7	15.2 ± 3.7	15.4 ± 4.0	16.1 ± 3.5
PUFA (% kcal/d)	5.2 ± 1.6	5.6 ± 1.7	4.8 ± 1.4	4.7 ± 1.4	5.7 ± 1.7
Trans fatty acid (% kcal/d)	0.4 ± 0.2	0.4 ± 0.2	0.3 ± 0.2	0.3 ± 0.2	0.4 ± 0.2
Total dietary fiber (g/d)	22.5 ± 9.8	19.2 ± 7.6	26.1 ± 11.4	26.3 ± 11.5	19.0 ± 7.5
Micronutrients					
Vitamin A (µg/d)	1973.0 ± 1450.3	1389.1 ± 867.1	2625.3 ± 1866.4	2548.4 ± 1886.9	1440.3 ± 926.8
Vitamin B1 (mg/d)	1.8 ± 0.6	1.7 ± 0.5	2.0 ± 0.6	1.9 ± 0.6	1.7 ± 0.5
Vitamin B2 (mg/d)	2.2 ± 0.7	2.0 ± 0.6	2.5 ± 0.8	2.3 ± 0.8	2.1 ± 0.6
Vitamin B3 (mg/d)	42.3 ± 11.8	40.7 ± 11.3	43.7 ± 12.4	42.9 ± 12.3	41.0 ± 11.6
Vitamin B6 (mg/d)	2.8 ± 0.9	2.4 ± 0.8	3.1 ± 1.0	3.1 ± 1.0	2.5 ± 0.8
Vitamin B12 (µg/d)	9.5 ± 4.9	9.1 ± 4.7	9.9 ± 5.4	9.7 ± 5.2	9.0 ± 4.7
Folic acid (µg/d)	408.8 ± 174.8	327.0 ± 122.9	501.1 ± 205.3	486.3 ± 204.5	335.7 ± 131.8
Vitamin C (mg/d)	280.5 ± 154.4	207.1 ± 100.2	362.1 ± 186.1	350.3 ± 184.0	216.8 ± 108.8
Vitamin D (µg/d)	6.3 ± 4.4	5.6 ± 3.8	6.9 ± 5.2	6.9 ± 5.1	5.6 ± 3.8
Vitamin E (mg/d)	7.0 ± 3.6	7.1 ± 3.7	6.9 ± 3.6	7.0 ± 3.8	7.0 ± 3.5
Calcium (mg/d)	1229.0 ± 469.6	1095.2 ± 422.1	1366.6 ± 504.3	1310.7 ± 516.8	1137.8 ± 421.3
Iron (mg/d)	17.1 ± 5.1	16.2 ± 4.6	18.0 ± 5.8	18.0 ± 5.7	16.1 ± 4.6
Potassium (mg/d)	4777.5 ± 1578.0	4152.0 ± 1223.7	5484.3 ± 1818.3	5322.1 ± 1825.9	4266.3 ± 1301.1
Sodium (mg/d)	3316.3 ± 2073.9	4090.2 ± 2622.5	2588.7 ± 1266.1	2545.0 ± 1110.5	4151.5 ± 2779.4
Magnesium (mg/d)	416.7 ± 123.7	385.3 ± 108.2	452.4 ± 138.6	448.5 ± 139.2	386.6 ± 109.3
Zinc (mg/d)	17.9 ± 10.9	15.4 ± 7.6	20.8 ± 13.8	20.0 ± 13.3	16.0 ± 8.7
Phosphorus (mg/d)	1931.1 ± 535.1	1818.9 ± 501.1	2044.0 ± 567.4	2005.2 ± 576.7	1844.6 ± 509.8
Selenium (µg/d)	95.4 ± 34.4	95.3 ± 35.9	94.3 ± 33.7	100.6 ± 36.3	88.9 ± 32.3
Food groups (g/day)					
Vegetables (g/d)	509.7 ± 328.9	366.3 ± 205.8	668.0 ± 410.1	652.9 ± 409.3	375.8 ± 223.9
Fresh Fruits (g/d)	347.4 ± 294.1	221.8 ± 163.6	494.4 ± 382.7	473.5 ± 370.4	235.6 ± 181.8
Nuts (g/d)	14.1 ± 25.7	11.7 ± 17.7	17.1 ± 33.7	19.2 ± 35.5	9.9 ± 15.4
Legumes (g/d)	22.8 ± 17.9	21.6 ± 16.1	24.2 ± 20.2	23.6 ± 18.2	21.9 ± 17.6
Whole grains (g/d)	13.2 ± 30.8	10.4 ± 30.8	15.4 ± 30.0	19.2 ± 38.1	8.4 ± 22.2
Refined grains (g/d)	58.6 ± 64.0	76.9 ± 77.1	38.7 ± 44.9	58.9 ± 70.1	56.6 ± 58.9
Fish and seafood (g/d)	97.2 ± 59.3	85.1 ± 50.4	109.3 ± 69.3	109.9 ± 67.8	82.8 ± 50.2
White meat (g/d)	46.9 ± 34.0	42.6 ± 30.5	50.6 ± 37.3	47.2 ± 36.1	45.8 ± 32.6
Red meat (g/d)	75.0 ± 45.4	80.0 ± 46.9	68.1 ± 43.8	66.2 ± 42.0	80.3 ± 48.0
Processed meat (g/d)	52.6 ± 34.3	62.3 ± 39.0	42.0 ± 28.1	39.5 ± 27.1	63.8 ± 38.5
Eggs (g/d)	23.4 ± 16.0	24.3 ± 16.3	22.1 ± 15.6	22.3 ± 15.5	24.1 ± 16.2
Low-fat dairy (g/d)	231.9 ± 246.6	143.8 ± 171.6	327.5 ± 294.1	291.3 ± 281.8	173.4 ± 202.8
Full-fat dairy (g/d)	157.5 ± 183.8	156.1 ± 154.4	152.0 ± 206.8	138.6 ± 186.5	166.2 ± 173.2
Olive oil (g/d)	15.1 ± 13.5	13.7 ± 13.4	15.9 ± 13.3	17.9 ± 14.6	12.4 ± 12.1
Other fats (g/d)	4.6 ± 11.2	5.2 ± 11.9	3.9 ± 10.0	4.2 ± 13.3	5.0 ± 9.7
Sweetened beverages (g/d)	64.7 ± 107.0	112.0 ± 154.2	26.5 ± 42.6	14.8 ± 23.7	132.1 ± 153.6

Bottled fruit juices (g/d)	23.7 ± 65.2	39.2 ± 94.7	10.3 ± 31.1	5.1 ± 15.6	48.3 ± 99.5
Sweets, cakes and pastry (g/d)	66.3 ± 48.6	79.2 ± 55.7	50.8 ± 37.8	46.8 ± 33.6	84.8 ± 59.0
Others					
Following a special diet (%)	1,145 (7.2%)	238 (4.5%)	576 (10.9%)	545 (10.3%)	259 (4.9%)
Snacking between meals (%)	5,396 (34.0%)	1,948 (36.8%)	1,622 (30.7%)	1,348 (25.5%)	2,238 (42.3%)
Supplement use, %	2,964 (18.7%)	864 (16.3%)	1,123 (21.2%)	1,026 (19.4%)	953 (18.0%)

For clarity and conciseness, only the extreme tertiles (T1 and T4) are presented to represent the contrast between the lowest and highest adherence to each dietary pattern. Total refers to the mean (SD) or n (%) for the entire sample. Percent Difference (Diff %) represents the relative change in intake between T3 and T1 for each specific dietary index, calculated as: [(T4 - T1) / T1] x 100. This metric evaluates the magnitude of the gradient within each pattern. Delta MUPF-UPF (p.p.) represents the difference in percentage points between the MUPF Diff% and the UPF Diff%. This comparison identifies which dietary index captures a wider range of variation (higher contrast) for a specific nutrient or food group within the study population. Abbreviations: SFA: Saturated Fatty Acids; MUFA: Monounsaturated Fatty Acids; PUFA: Polyunsaturated Fatty Acids; MUPF: Minimally or unprocessed food; UPF: Ultraprocessed food.

Supplementary Table S3. Hazard Ratios (95% CI) for the association between the relative energy intake from minimally or unprocessed foods (MUPF) and ultra-processed foods (UPF) (% of kcal/day) and the self-reported diagnosed incidence of immune-mediated inflammatory diseases (IMIDs). SUN Project.

	**Tertiles of percentual of UPF kcal consumption (% kcal/day)**				**Tertiles of percentual of MUPF kcal consumption (% kcal/day)**			
	**T1 (<20.6%)**	**T2 (20.6-28.7%)**	**T3 (>28.7%)**	*p-trend*	**T1 (<41.8%)**	**T2 (41.8-50.7%)**	**T3 (>50.7%)**	*p-trend*
**Immune-mediated inflammatory diseases**								
Persons-Year	74,941	75,899	76,484		77,717	76,466	73,140	
Cases/N	107/5,292	88/5,291	103/5,291		108/5,292	105/5,291	85/5,291	
Unadjusted	Ref	1.01 (0.76-1.34)	1.37 (1.04-1.82)	**0.030**	Ref	0.96 (0.73-1.25)	0.71 (0.53-0.95)	**0.021**
Age and sex adjusted	Ref	1.04 (0.78-1.38)	1.45 (1.09-1.93)	**0.012**	Ref	0.93 (0.71-1.22)	0.64 (0.48-0.85)	**0.002**
Multivariable adjusted	Ref	1.01 (0.75-1.34)	1.35 (1.01-1.81)	**0.045**	Ref	0.96 (0.73-1.26)	0.68 (0.50-0.92)	**0.014**
**Psoriasis**								
Cases/N	57/5,292	47/5,291	61/5,291		66/5,292	56/5,291	43/5,291	
Unadjusted	Ref	0.90 (0.61-1.33)	1.25 (0.86-1.82)	0.234	Ref	0.85 (0.59-1.21)	0.64 (0.44-0.95)	**0.025**
Age and sex adjusted	Ref	0.95 (0.64-1.40)	1.33 (0.91-1.94)	0.142	Ref	0.85 (0.59-1.21)	0.59 (0.40-0.87)	**0.008**
Multivariable adjusted	Ref	0.91 (0.62-1.36)	1.23 (0.84-1.82)	0.282	Ref	0.86 (0.60-1.24)	0.63 (0.42-0.95)	**0.029**
**Rheumatoid arthritis**								
Cases/N	47/47	35/5,291	37/5,291		38/5,292	42/5,291	39/5,291	
Unadjusted	Ref	1.05 (0.68-1.64)	1.46 (0.94-2.28)	0.108	Ref	1.07 (0.69-1.66)	0.83 (0.53-1.30)	0.401
Age and sex adjusted	Ref	1.08 (0.69-1.69)	1.58 (1.00-2.48)	0.059	Ref	1.01 (0.65-1.57)	0.70 (0.44-1.11)	0.119
Multivariable adjusted	Ref	1.05 (0.67-1.64)	1.45 (0.92-2.30)	0.126	Ref	1.05 (0.67-1.64)	0.75 (0.46-1.21)	0.221
**Vitiligo**								
Cases/N	8/5,292	7/5,291	9/5,291		8/5,292	11/5,291	5/5,291	
Unadjusted	Ref	1.11 (0.40-3.10)	1.65 (0.62-4.43)	0.320	Ref	1.34 (0.54-3.34)	0.55 (0.18-1.69)	0.326
Age and sex adjusted	Ref	1.10 (0.39-3.10)	1.64 (0.60-4.47)	0.333	Ref	1.27 (0.51-3.19)	0.51 (0.16-1.59)	0.262
Multivariable adjusted	Ref	0.98 (0.34-2.77)	1.32 (0.47-3.67)	0.596	Ref	1.30 (0.52-3.30)	0.56 (0.18-1.81)	0.371

Hazard ratios (HR) and 95% confidence intervals (CI) compare upper tertiles (T2–T3) vs. the lowest tertile (T1) of dietary consumption. All models are adjusted for sex, college education, smoking status, body mass index, following a special diet, snacking between meals, total physical activity, time watching television, duration of nighttime sleep and alcohol consumption. Abbreviations: HR, hazard ratio; CI, confidence interval.

Supplementary Table S4. Hazard Ratios (95% CI) for the association between the absolute energy intake of minimally or unprocessed foods (MUPF) and ultra-processed foods (UPF) (kcal/day) with the self-reported diagnosed incidence of immune-mediated inflammatory diseases (IMIDs). The SUN Project.

	**Tertiles of total MUPF kcal consumption (kcal/day)**				**Tertiles of total UPF kcal consumption (kcal/day)**			
	**T1 (<971.3 kcal/d)**	**T2 (971.3-1168.4 kcal/d)**	**T3 (>1168.4 kcal/d)**	***p-trend***	**T1 (<437.4 kcal/d)**	**T2 (437.4-692.5 kcal/d)**	**T3 (>692.5 kcal/d)**	***p-trend***
**Immune-mediated inflammatory diseases**								
Persons-Year	77,847	76,125	73,352		73,565	76,642	77,118	
Cases/N	108/5,292	102/5,291	88/5,291		95/5,292	111/5,291	92/5,291	
Unadjusted	Ref	0.92 (0.70-1.20)	0.74 (0.56-0.98)	**0.037**	Ref	1.40 (1.06-1.85)	1.29 (0.96-1.72)	0.082
Age and sex adjusted	Ref	0.88 (0.67-1.15)	0.66 (0.50-0.88)	**0.005**	Ref	1.50 (1.14-1.98)	1.39 (1.03-1.87)	**0.024**
Multivariable adjusted	Ref	0.95 (0.72-1.25)	0.72 (0.53-0.97)	**0.029**	Ref	1.37 (1.02-1.84)	1.16 (0.81-1.67)	0.387
**Psoriasis**								
Cases/N	65/5,292	49/5,291	51/5,291		40/5,292	73/5,291	52/5,291	
Unadjusted	Ref	0.75 (0.52-1.09)	0.78 (0.54-1.12)	0.166	Ref	1.94 (1.32-2.87)	1.47 (0.96-2.23)	0.087
Age and sex adjusted	Ref	0.73 (0.50-1.06)	0.72 (0.50-1.05)	0.086	Ref	2.11 (1.43-3.12)	1.60 (1.04-2.45)	**0.033**
Multivariable adjusted	Ref	0.79 (0.54-1.15)	0.80 (0.54-1.18)	0.244	Ref	1.98 (1.31-2.99)	1.40 (0.84-2.35)	0.220
**Rheumatoid arthritis**								
Cases/N	40/5,292	45/5,291	34/5,291		52/5,292	30/5,291	37/5,291	
Unadjusted	Ref	1.06 (0.69-1.62)	0.68 (0.43-1.09)	0.106	Ref	0.80 (0.51-1.26)	1.17 (0.76-1.80)	0.567
Age and sex adjusted	Ref	0.98 (0.64-1.50)	0.57 (0.35-0.91)	**0.017**	Ref	0.86 (0.55-1.36)	1.27 (0.82-1.97)	0.351
Multivariable adjusted	Ref	1.08 (0.69-1.67)	0.60 (0.37-0.98)	**0.038**	Ref	0.77 (0.48-1.25)	1.05 (0.61-1.81)	0.949
**Vitiligo**								
Cases/N	7/5,292	11/5,291	6/5,291		6/5,292	11/5,291	7/5,291	
Unadjusted	Ref	1.50 (0.58-3.88)	0.76 (0.25-2.28)	0.629	Ref	2.23 (0.81-6.09)	1.61 (0.53-4.91)	0.388
Age and sex adjusted	Ref	1.41 (0.54-3.67)	0.72 (0.24-2.17)	0.551	Ref	2.27 (0.83-6.22)	1.66 (0.54-5.13)	0.360
Multivariable adjusted	Ref	1.56 (0.59-4.15)	0.78 (0.25-2.44)	0.685	Ref	1.91 (0.67-5.47)	1.08 (0.29-4.03)	0.904

Hazard ratios (HR) and 95% confidence intervals (CI) compare upper tertiles (T2–T3) vs. the lowest tertile (T1) of dietary consumption. All models are adjusted for sex, college education, smoking status, body mass index, following a special diet, snacking between meals, total physical activity, time watching television, duration of nighttime sleep, alcohol consumption, and total daily energy intake (as a covariate in adjusted model). Abbreviations: HR, hazard ratio; CI, confidence interval.

Supplementary Table S5. Hazard Ratios (95% CI) for the association between the absolute weight intake (grams/day) of minimally processed foods (MUPF) and ultra-processed foods (UPF) and the self-reported diagnosed incidence of immune-mediated inflammatory diseases (IMIDs). The SUN Project.

	**Tertiles of MUPF (gram consumption/day)**				**Tertiles of percentual of UPF (gram consumption/day)**			
	**T1 (<1394.0g/d)**	**T2 (1394.0-1822.6g/d)**	**T3 (>1822.6g/d)**	***p-trend***	**T1 (<200.3g/d)**	**T2 (200.3-305.9g/d)**	**T3 (>305.9g/d)**	***p-trend***
**Immune-mediated inflammatory diseases**								
Persons-years	77,723	75,730	73,870		76,093	75,854	75,377	
Cases/N	120/5,292	94/5,291	84/5,291		102/5,292	104/5,291	92/5,291	
Unadjusted	Ref	0.74 (0.56-0.96)	0.62 (0.47-0.83)	**0.001**	Ref	1.32 (1.00-1.74)	1.38 (1.03-1.84)	**0.028**
Age and sex adjusted	Ref	0.70 (0.53-0.93)	0.57 (0.43-0.77)	**<0.001**	Ref	1.33 (1.01-1.76)	1.34 (0.99-1.81)	**0.047**
Multivariable adjusted	Ref	0.76 (0.57-1.00)	0.61 (0.45-0.83)	**0.001**	Ref	1.35 (1.02-1.80)	1.29 (0.95-1.75)	0.092
**Psoriasis**								
Cases/N	76/5,292	43/5,291	46/5,291		51/5,292	62/5,291	52/5,291	
Unadjusted	Ref	0.56 (0.38-0.81)	0.59 (0.41-0.85)	**0.003**	Ref	1.38 (0.95-2.01)	1.26 (0.85-1.88)	0.245
Age and sex adjusted	Ref	0.55 (0.37-0.80)	0.57 (0.39-0.84)	**0.003**	Ref	1.38 (0.94-2.01)	1.20 (0.80-1.80)	0.380
Multivariable adjusted	Ref	0.59 (0.40-0.87)	0.62 (0.42-0.93)	**0.014**	Ref	1.41 (0.96-2.09)	1.15 (0.76-1.75)	0.512
**Rheumatoid arthritis**								
Cases/N	42/5,292	43/5,291	34/5,291		43/5,292	42/5,291	34/5,291	
Unadjusted	Ref	0.90 (0.59-1.37)	0.63 (0.40-1.00)	0.050	Ref	1.48 (0.96-2.27)	1.62 (1.02-2.58)	**0.033**
Age and sex adjusted	Ref	0.83 (0.54-1.28)	0.53 (0.33-0.85)	**0.009**	Ref	1.56 (1.01-2.41)	1.67 (1.03-2.69)	**0.027**
Multivariable adjusted	Ref	0.88 (0.57-1.37)	0.55 (0.34-0.90)	**0.017**	Ref	1.56 (1.00-2.43)	1.59 (0.98-2.58)	**0.048**
**Vitiligo**								
Cases/N	6/5,292	11/5,291	7/5,291		11/5,292	4/5,291	9/5,291	
Unadjusted	Ref	1.71 (0.63-4.63)	1.04 (0.35-3.10)	0.991	Ref	0.47 (0.15-1.50)	1.24 (0.49-3.12)	0.733
Age and sex adjusted	Ref	1.60 (0.58-4.39)	0.94 (0.30-2.93)	0.861	Ref	0.46 (0.14-1.46)	1.13 (0.44-2.92)	0.872
Multivariable adjusted	Ref	1.76 (0.63-4.91)	1.15 (0.36-3.66)	0.837	Ref	0.43 (0.13-1.41)	1.00 (0.37-2.65)	0.968

Hazard ratios (HR) and 95% confidence intervals (CI) compare upper tertiles (T2–T3) vs. the lowest tertile (T1) of dietary consumption. All models are adjusted for sex, college education, smoking status, body mass index, following a special diet, snacking between meals, total physical activity, time watching television, duration of nighttime sleep, alcohol consumption, and total daily energy intake (as a covariate in adjusted model). Abbreviations: HR, hazard ratio; CI, confidence interval.

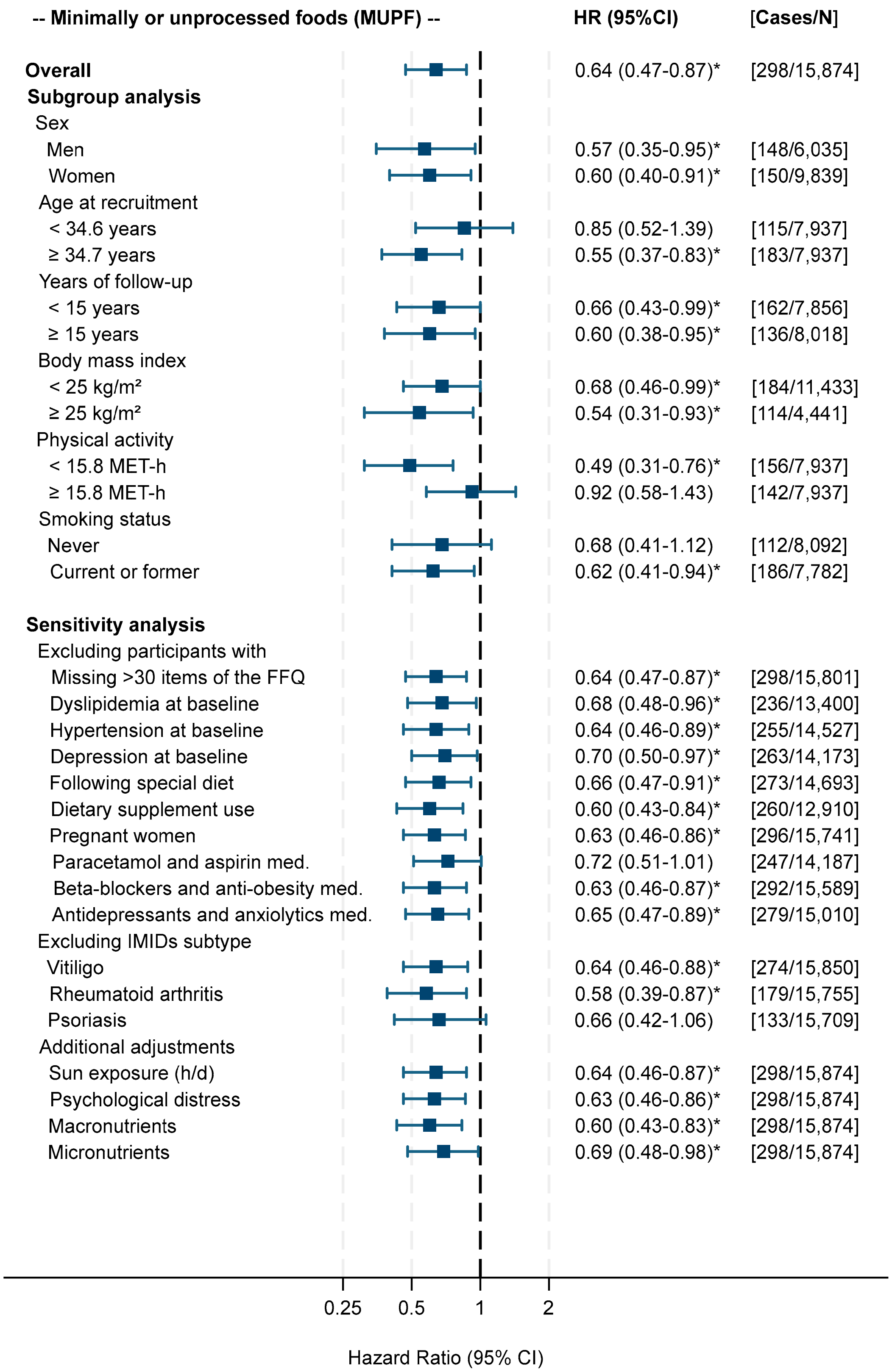

Supplementary Figure S1. Forest plot of stratified subgroup analyses and sensitivity analyses for the association between consumption of minimally or unprocessed foods (MUPF) and the risk of immune-mediated inflammatory diseases (IMIDs). The SUN Project.

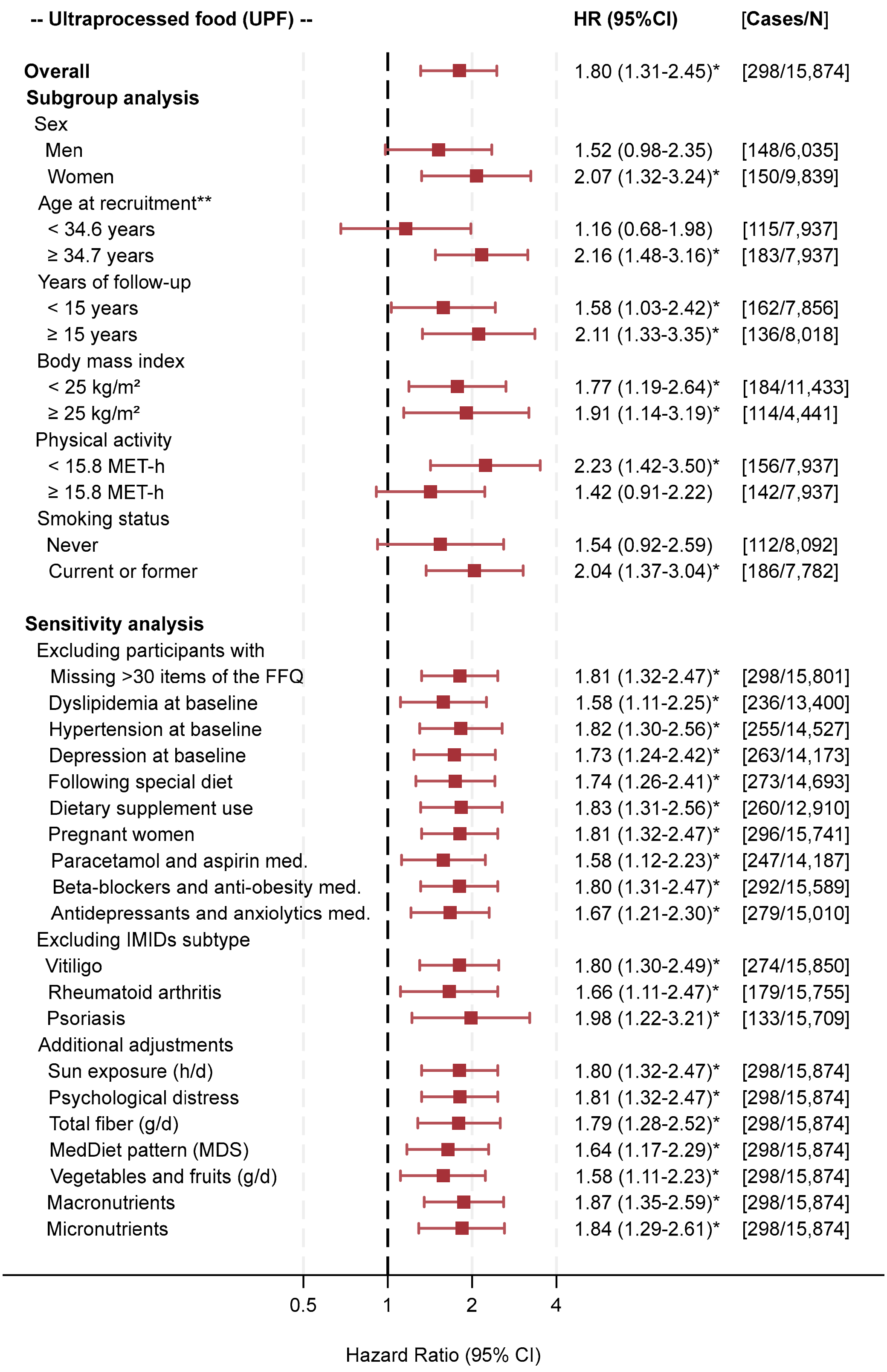

Supplementary Figure S2. Forest plot of stratified subgroup analyses and sensitivity analyses for the association between consumption of ultraprocessed foods (UPF) and the risk of immune-mediated inflammatory diseases (IMIDs). The SUN Project.
